# Visualizing advances in the future of primate neuroscience research

**DOI:** 10.1016/j.crneur.2022.100064

**Published:** 2022-12-13

**Authors:** Peter Janssen, Tadashi Isa, Jose Lanciego, Kirk Leech, Nikos Logothetis, Mu-Ming Poo, Anna S. Mitchell

**Affiliations:** a Laboratory for Neuro- and Psychophysiology, KU Leuven, Belgium; b Graduate School of Medicine, Kyoto University, Japan; c Department Neurosciences, Center for Applied Medical Research (CIMA), University of Navarra, CiberNed., Pamplona, Spain; d European Animal Research Association, United Kingdom; e International Center for Primate Brain Research, Shanghai, China; f School of Psychology, Speech and Hearing, University of Canterbury, Christchurch, New Zealand; g Department of Experimental Psychology, University of Oxford, United Kingdom

**Keywords:** Gene therapy, Viral vector approaches, Transgenics, CRISPR/Cas9, Gene editing

## Abstract

Future neuroscience and biomedical projects involving non-human primates (NHPs) remain essential in our endeavors to understand the complexities and functioning of the mammalian central nervous system. In so doing, the NHP neuroscience researcher must be allowed to incorporate state-of-the-art technologies, including the use of novel viral vectors, gene therapy and transgenic approaches to answer continuing and emerging research questions that can only be addressed in NHP research models. This perspective piece captures these emerging technologies and some specific research questions they can address. At the same time, we highlight some current caveats to global NHP research and collaborations including the lack of common ethical and regulatory frameworks for NHP research, the limitations involving animal transportation and exports, and the ongoing influence of activist groups opposed to NHP research.

## Highlights

• Neuroscience research involving non-human primate models remains essential.
• State-of-the-art technologies are rapidly evolving for use in non-human primate models.
• Non-human primate models allow specific neuroscience research questions to be answered.

## 1 Background

The use of non-human primates (NHPs) in biomedical research has been extensively evaluated in Europe (see for example the Weatherall report (Weatherall Report, 2006). Initiated by the European Commission, the Scientific Committee on Health, Environmental and Emerging Risks (SCHEER) adopted an opinion on the use of NHPs in 2009 and provided an update of this report in 2017 (SCHEER, 2017). In the first SCHEER report, the use of NHPs was considered essential for pharmaceutical developments, research on infectious diseases, (xeno)transplantation and neuroscience. In the same vein, the SCHEER committee of 2017 concluded that several important factors contribute to the continued need for NHPs at least in the aforementioned domains.

For example, safety testing of pharmaceuticals and medical devices most often require NHPs because of the poor definition of pharmacokinetic parameters in isolated *in vitro* systems, and difficulties in extrapolating from *in vitro* data to humans. Animal models (including NHPs) are not able to encapsulate a complete disease state present in humans, so there remain some difficulties in extrapolating from animal models to humans as well. Nevertheless, with respect to infectious diseases, the committee was remarkably prescient given their statement that ‘it is unlikely that new technologies will negate the need for infectious NHP models in the near future due to emerging and re-emerging pathogens’, which proved to be more than justified in the Covid-19 pandemic.

In neuroscience – and in systems neuroscience in particular - important limitations continue to exist. Functional imaging experiments in human volunteers, for instance, while non-invasive and currently an easy to implement methodology, in actual fact, only provide indirect measurements of neural population activity at very low spatio-temporal resolution. The limitations of the functional Magnetic Resonance Imaging (fMRI) are not due to physics or poor engineering, and are unlikely to be resolved by increasing the sophistication and power of the scanners; they are instead due to the circuitry and functional organization of the brain. The fMRI signal cannot differentiate between function-specific processing and neuromodulation, between bottom-up and top-down signals, and it may occasionally confuse excitation and inhibition (Logothetis, 2008; Logothetis and Wandell, 2004). The magnitude of the fMRI signal cannot be quantified to accurately reflect differences between brain regions, or between tasks within the same region. The origin of the latter problem is not our current inability to accurately estimate cerebral metabolic rate (CMRO2) from the blood-oxygen-level-dependent (BOLD) imaging signal, but the fact that hemodynamic responses are sensitive to the size of the activated population, which may change as the sparsity of neural representations varies spatially and temporally. Observations made by means of functional imaging – barring those related to structural changes or regional tissue-damage – provide potential explanations, the selective verification of which most often requires concurrent or subsequent invasive studies (Logothetis, 2003, 2008; Logothetis and Wandell, 2004).

To better understand the relation of the BOLD imaging signal to its underlying neural events, we briefly review the nature of the neurophysiological signal commonly reported from invasive animal studies (for extensive reviews see Logothetis, 2008; Logothetis and Wandell, 2004). The comprehensive (with a bandwidth of 0.05Hz through 23–30 kHz) extracellular field potential measured in intracranial recordings captures at least three different types of activity: single-unit activity representing the action potentials of well isolated neurons next to the electrode tip; multiple unit activity (MUA) reflecting the spiking of small neural populations in a sphere of 100–300 μm radius; and perisynaptic activity of a neural population within 0.5–3 mm of the electrode tip, which is reflected in the variation of the low-frequency components of the extracellular field potential. MUA and local field potentials (LFPs) can be reliably segregated by frequency band separation. The frequency range of 800–3,000 Hz is used in most recordings to obtain MUA; a low-pass filter cutoff of approximately 250 Hz is used to obtain LFP. A large number of experiments have presented data indicating that such a band separation does indeed underlie different neural activities. Thus, their relationship to the BOLD imaging signal is not only able to provide us with insights into the mechanisms underlying the hemodynamic changes, it can also help us with a more detailed interpretation of the functional significance of the activation patterns observed in MR imaging.

Combined physiology-fMRI experiments showed that the BOLD imaging signal predominantly reflects regional *perisynaptic* activity, which includes the classical events of synaptic transmission with its respective population of excitatory or inhibitory postsynaptic potentials, as well as a number of integrative processes, including somatic and dendritic spikes with their ensuing afterpotentials, and voltage-dependent membrane oscillations. These events represent the regional modulation, processing and elaboration of incoming signals, and correlate largely with changes in the LFPs. In other words, the LFPs reflect effects such as neuromodulation, the influence of “background” barrages of synaptic potentials on excitation-inhibition balance, and interactions between interneurons and pyramidal cells, all of which might act as determinant factors for the ensuing hemodynamic response (Logothetis, 2008). As long as spiking and LFP activity resemble each other (e.g., during feedforward propagation of sensory signals), the BOLD response appears to be strongly correlated with both signals. Yet a striking, undiminished hemodynamic response may often be observed in cases in which neuronal firing is entirely absent despite a clear and strong stimulus-induced modulation of the field potentials (Mathiesen et al., 1998; Rauch et al., 2008; Viswanathan and Freeman, 2007). Such results were later confirmed in a series of experiments using either mass-univariate linear and nonlinear methods (Lippert et al., 2010; Lüdtke et al., 2010; Zappe et al., 2008), or multivariate techniques (Biessmann et al., 2010; Murayama et al., 2010).

Despite its shortcomings, fMRI is an outstanding tool for gaining insights into network activity, and it can often reveal activated regions that may be missed by electrical recordings. Activation of populations whose cells have a close-field (low dipole moment) arrangement may occasionally be missed both in the LFP and MUA signals; yet changes in energy metabolism and subsequent changes in hemodynamics may still impact the fMRI signal. Importantly, activations during imaging are always neurogenic and maps of activity reveal active networks, regardless of whether they reflect sensory or modulatory signals (Logothetis, 2010; Logothetis et al., 2010). Interpreting and understanding neuroimaging signals requires complementary invasive neuroscience research.

Yet, invasive studies in humans remain rare (e.g., Quiroga et al., 2005; Hochberg et al., 2006), and although in recent years more human cortical areas have been explored (Aflalo et al., 2015; Decramer et al., 2019), the number of areas that can be recorded in humans remains extremely limited. Moreover, *in vitro* approaches to study disease processes have important limitations due to the absence of a blood-barrier, and immune and vascular systems. The SCHEER committee correctly concluded that it is ‘difficult to predict a timetable for complete replacement for each of the research areas’.

In parallel, we are witnessing a surge in the application of novel research techniques that were developed over many years in rodents, and are now being progressively transferred to NHPs, some of which are discussed below, and include: optogenetic interventions (De et al., 2020; Tye and Deisseroth, 2012), calcium imaging (Tang et al., 2020), viral vector technology and stem cell therapy (Kikuchi et al., 2017), and high density electrophysiology, e.g., as made possible by Neuropixels® probes (Steinmetz et al., 2021). All these examples of very powerful neuroscience research techniques are becoming available to study the NHP brain to advance the translation of basic research findings acquired in animals to the human brain.

It is also worth noting that despite all the advances being made in neuroscience research in the past decades, we are still lacking disease-modifying therapeutics. For instance, due to the foresight of Juan Carlos López, 140 experts were invited to a round-table discussion held in Germany to identify the main obstacles to translational research in neurodegeneration (López, 2010). As a result of this initiative at least five main bottlenecks were identified, such as (1) the need to learn more about the biology of disease, (2) the need for developing better animal models, (3) the availability of better biomarkers, (4) the design of better preclinical and clinical trials, and (5) to invest more in infrastructures and human resources. It is without doubt that neuroscience research, when conducted in NHPs in particular, is best suited for addressing several of these unmet needs, of particular relevance when gaining more insight on disease biology, setting up better animal models, testing drug candidates in preclinical stages and brain-computer interface (BCI) technologies. In other words, and although at a greater expense, NHP research in the fields of neuroscience remains fundamental to further minimize clinical trial failures, pushing forward true translational neuroscience efforts.

## 2 NHP models remain fundamental for neuroscience and biomedical research

We have learned a great deal from neuroanatomical, neurophysiological, direct electrical- and optical-stimulation, molecular and lesion studies in NHPs (mainly macaques). Along with other species, primates were also routinely used to develop and test treatments for diseases and medical conditions. Examples include the polio vaccine, insulin treatments, safer techniques for heart, eye, and bone surgeries, substantially better medical care for prenatal and postnatal infants, treatments for polycystic ovary syndrome and endometriosis in women, discovery of the Rh blood incompatibility in infants and mothers, and many more (see, for instance, MedlinePlus and sites of National Primate Research Centers, e.g. https://nprcresearch.org/primate/).

Yet, translational NHP studies have not been the only type of research to contribute to medical progress. Fundamental basic, curiosity-driven research substantially improved our understanding of brain function and dysfunction. A striking example is the brain research that preceded so-called Constraint-Induced Movement Therapy (CIMT) that was developed in NHPs by Edward Taub (Taub et al., 2002). Deep Brain Stimulation (DBS) used in the treatment of patients with Parkinson's Disease (PD) is another example of basic research influencing an applied medical one. The method was developed from research on macaques (Rosenow et al., 2004). The background information collected by curiosity driven, basic research is of fundamental importance for any rising question in any applied research field. Moreover, basic research in primates offered the first insights into the hierarchical organization of the visual system, each stage of which consists of different areas or modules that work - to some extent - parallel to each other, analyzing different visual attributes (Felleman and Van Essen, 1991; Hubel, 1988; Livingstone and Hubel, 1988; Zeki, 1993, 1998). Selective damage to different areas demonstrated that the dorsal (parietal cortex) stream processes information about the location and motion of objects, while the ventral (temporal cortex) stream processes information related to object recognition (Mishkin et al., 1983; Ungerleider and Mishkin, 1982).

Neurophysiological studies in the NHP have also offered insights into the neural underpinnings of the processes of attention, decision making (Desimone and Duncan, 1995; Gold and Shadlen, 2007; O'Connell et al., 2018; Sugrue et al., 2005), higher order planning (Rushworth and Behrens, 2008), conscious perception of multistable objects (Logothetis, 1999), and object recognition (DiCarlo and Cox, 2007; DiCarlo et al., 2012; Logothetis, 1998). Remarkably, non-human primates were also the main source of information related to cognitive capacities, usually associated only with humans, including cultural transmission, and the origin of language (Lloyd, 2004). Overall, NHP studies like those very briefly described above, offer true insights into the cognitive capacities of humans, likely also because of the anatomical and functional similarities of the two species.

The many differences in motor and cognitive behavior between rodents and primates have been well documented. These differences in capacities are supported by alterations in the central nervous system (CNS). In essence, our CNS is not simply a larger version of the rodent. Major areas of the brain exist in primates that are absent in rodents. For example, it has long been known that rodents lack a so called direct corticomotoneuronal (CM) pathway for upper limb control, that is present in humans, great apes and some monkeys, and plays a major role in dexterous hand movements. The CM pathway is indeed thought to underly our ability to manufacture and use tools, and even play a music instrument. But, this additional connection is not the only major difference between rodent and primate in terms of the cortical control of movement. The primate cerebral cortex contains at least 4 cortical motor areas on the medial wall of each hemisphere that appear to be entirely absent in the rodent. These cortical areas have neurons in layer 5 with axons that descend to the spinal cord to make synaptic connections with last order spinal interneurons that drive motoneuron activity. Thus, the number and extent of the cortical areas that control movement is massively expanded in primates compared to rodents (Strick et al., 2021). A similar expansion in the number of cortical areas involved in descending control of autonomic function also occurs in primates relative to rodents (Dum et al., 2019).

The addition and expansion of circuits is not limited to the cerebral cortex, but is seen in other brain regions as well. One striking example is the ventral portion of the dentate nucleus in the cerebellum (Strick et al., 2009). The ventral dentate is interconnected with regions of prefrontal cortex and is linked to cognitive rather than motor function. This portion of the dentate is greatly expanded in NHPs and displays its greatest enlargement in great apes and humans. In addition, given the many similarities in primate brain structure and function, NHPs are the most relevant model for understanding complex brain diseases such as Alzheimer's and Parkinson's disease (PD), or various brain cancer types, such as the Acute Myeloid Leukemia, and Glioblastoma multiforme (GBM), one of the most frequently occurring tumors in the CNS and the most malignant tumor among gliomas. It follows, that if one of our goals is to make discoveries that will not only increase the dearly needed fathoming into the state and function of brain systems and subsystems, but will ultimately also improve the human life-conditions and create new treatments and cure for diseases of the nervous system, then NHP research is vital and indispensable.

Evidently, for an external observer, it is extremely difficult to evaluate in which direction the use of NHPs in biomedical research should or will go. On the one hand, researchers worldwide have been developing innovations to replace NHPs, when possible. For example, the production of the polio vaccine required large numbers of macaques in the past, but is now replaced by cell cultures. Further, many researchers nowadays study the visual cortex in mice instead of in NHPs. However, there are limitations in translating results from rodent models to primates given the species differences in visual abilities (e.g., differences in binocular disparity and color spectrum detection), differences in cortical size, and the relative reliance on vision for primates verses other sensory processes in rodents (Graïc et al., 2022; Halley and Krubitzer, 2019; Laramée and Boire, 2015). In addition, biomedical researchers investigating human retinal damage and treatments still require NHP models given the similarities in the vascular supply and in the retina (e.g. the presence of a fovea as in humans) of NHPs and humans.

Moreover, new scientific questions continuously emerge, where a relevant technology may not yet be available, and testing in NHPs may be the only valid path to take. For example, invasive BCIs have been developed in NHPs to help paralyzed patients regain mobility and autonomy, and new BCI approaches may one day help blind people to see again with high resolution (Chen et al., 2020). Moreover, the large brains of Old World Macaques with sulci and gyri similar to the human brain, makes them highly suitable to study the neural effects of several noninvasive neuromodulation techniques, which are widely used in human volunteers but poorly understood at the neuronal level (Romero et al., 2019). These are just a few examples of new research avenues for which the NHPs remain essential, and it is reasonable to assume that new scientific questions that can only be addressed in NHPs will continue to emerge in the foreseeable future.

Research in NHPs is crucial also in the development of drugs and therapeutics. Given their phylogenetic similarity with humans, NHPs are indispensable for the development and validation of drugs and therapeutics. In line with regulations in nearly all countries worldwide, all new drugs must be tested on a rodent and non-rodent species before receiving approval. In some cases, NHPs are the most appropriate species for testing of new medicines, thanks in part to their evolutionary similarity to humans. Examples include testing for ocular treatments, reproductive health, cardiovascular safety evaluations, and monitoring the metabolism of new drugs during safety testing.

The use of NHPs is particularly important in the development of biotherapeutic drugs, such as monoclonal antibody treatments, peptide molecules, hormones, and gene therapies. Biotherapeutics are often targeted towards very specific human sequences. When other animal species are used for testing these drugs, the animal will often develop antibodies against the drug itself, making it impossible to understand the activity of the molecule amongst potential off-target effects (Yu et al., 2014). Given the close genetic similarity between NHPs and humans, it is far less likely that cross-reactions will occur, making NHPs the most suitable model for testing in these cases.

## 3 Novel viral vector approaches

New viral vector technology has made it possible to manipulate specific pathways in the brain at an unprecedented scale, which has clarified their role in, for example, recovery after nervous system injuries. The intersectional approach for the pathway-selective manipulation of neural circuits by combining two viral vectors was first introduced by Kinoshita et al. (2012). This novel method was used for perturbing the transmission of propriospinal neurons (PNs) in the mid-cervical spinal cord (C3-C4 PNs) in macaques. Previously it had been established that C3-C4 PNs play a major role in the control of forelimb reaching in cats, which have no direct connection between the motor cortex and spinal motoneurons (Alstermark and Lundberg, 1992). However, their functions were still unclear in the primates, which have the aforementioned CM pathway, optimizing and fine-tuning their hand movements. About 10 years ago, the pathway-selective manipulation in rodents always required the generation of a transgenic line, which was not realistic in primates. Consequently, Kinoshita and colleagues used two viral vectors for pathway-selective manipulation, one vector specified for retrograde transport injected into the projection area and another vector injected into the location of the cell bodies of C3-C4 PNs. After waiting two months, the administration of doxycycline (Dox) to the monkeys induced deficits in precision grip and/or reaching movements, while electrophysiological experiments demonstrated that the transmission through the C3-C4 PNs was blocked by approximately 90%. Now such double viral vector techniques are very commonly used in rodents by a variety of retrograde and anterograde vectors (Wahl et al., 2014; Pocratsky et al., 2017), but interestingly the first success of this modern technique was achieved in a NHP model as transgenic animals were not available. After Kinoshita et al. (2012), the technique has been applied in several other NHP models. For example, (1) in the same C3-C4 PNs in monkeys with spinal cord injury to demonstrate their functions in recovery after spinal cord injury (Tohyama et al., 2017); (2) to the superior colliculus-to-pulvinar pathway to demonstrate the role of the pathway in saccade control in blindsight monkeys after the primary visual cortex was lesioned (Kinoshita et al., 2019); (3) to the pathway from the ventral tegmental area to the nucleus accumbens to demonstrate the role of this pathway in the control of motivation (Vancraeyenest et al., 2020); and (4) to block the cortico-cortical pathway between the premotor cortex and medial prefrontal cortex to perturb social cognition in monkeys (Ninomiya et al., 2020).

Another recent trend in primate neuroscience research is the introduction of systems neuroscience approaches to model neuronal injuries or neuropsychiatric disorders in animals to clarify the underlying neuronal mechanisms contributing to the deficits, and to develop therapeutic strategies for recovery. Lesion models in NHPs are not new, but classical studies were primarily focused on behavioral analyses, which clarified changes in normal behavioral and cognitive functioning as a consequence of removal (lesion) of a brain structure (Klink et al., 2021). Here, modern techniques for recording and analyzing neuronal signals were not always applied. However, as technology has advanced, many neuroscientists are now combining state-of-the-art perturbation methods, neuroimaging, and neurophysiological recordings with behavioral and cognitive analyses (for review, see Klink et al., 2021) to further investigate and advance our understanding about the underlying neuronal signals that are contributing to brain injuries or neuropsychiatric disorders.

Furthermore, recent studies on spinal cord injuries have focused on the recovery process and its mechanism. In these studies, a variety of techniques spanning from whole brain neuroimaging with positron emission tomography (PET), electrophysiology using electrocorticography (ECG) in behaving states, intracellular recordings in terminal experiments under anesthesia, behavioral analysis combined with electromyography (EMG) recordings, pharmacological manipulation and circuit manipulation with viral vectors and neuroanatomical inspections are combined (see Isa, 2019). Similarly, in a series of studies to clarify the neuronal mechanism of blindsight, combinations of techniques including computational modeling of saliency maps and signal detection theory were applied to clarify the cognition in the macaque model (for review, see Isa and Yoshida, 2021). In these cases, such analyses can never be applied to human patients, and for now, this analysis method is still mostly limited to rodent models, which have limitations in extrapolation to human diseases. If such translational studies with sophisticated analytical techniques are applied for the analysis of genome-edited NHP models, it will be a promising field for primate neuroscience studies in the future.

## 4 Novel gene therapy approaches that model diseases and therapeutics

At present, neuroscientists are privileged witnesses of the spectacular rise of modern molecular-genetic techniques based on the use of modified viruses as vectors carrying a given genetic material for transduction of different CNS cellular populations, including neurons and glial cells. By taking advantage of viral vectors, we are facing a quickly changing scenario moving forward into two main directions. First, strong efforts are ongoing in an attempt to develop and characterize animal models better mimicking human neurodegenerative disorders. Second, viral vectors carrying different genetic payloads are currently undergoing extensive preclinical and clinical testing. Indeed, to push forward translational preclinical initiatives based on gene therapies, the use of NHP models obviously plays an instrumental role.

## 5 Viral vectors for disease-modeling purposes

Although neurotoxin-based mammalian animal models of PD have settled the basis for most of our current understanding of basal ganglia function and dysfunction (Lanciego et al., 2012), these models failed to recapitulate the main neuropathological hallmarks that typically characterize PD (e.g. dopaminergic cell death driven by alpha-synuclein (αSyn) aggregation). Most importantly, the acute dopaminergic damage induced by neurotoxins represents an important limitation when testing neuroprotective, disease-modifying approaches. The discovery that αSyn is the main component of Lewy bodies (Spillantini et al., 1997) drastically changed the field of animal modeling in PD. Accordingly, several murine transgenic lines overexpressing different mutated or wild type forms of αSyn have been made available, most of these models reproducing several key features of PD. Although these transgenic mouse lines are appealing choices for testing new therapeutic candidates against αSyn aggregation, it is worth noting that in most cases neuronal loss in the substantia nigra pars compacta (SNpc) is often weak or even absent (Visanji et al., 2016). In summary, most of the available transgenic mice for αSyn aggregation are lacking the appropriate phenotype. Therefore, at present there is a clear trend for the development and characterization of NHP (macaque) models reproducing the progressive dopaminergic neuronal degeneration as a result of αSyn aggregation.

The use of recombinant adeno-associated viral vectors (rAAVs) coding for αSyn for the purpose of PD modeling in NHPs currently is in the spotlight. Indeed, rAAVs are versatile enough to collectively represent an appropriate choice for disease modeling. When designed this way, the intraparenchymal delivery of different rAAV serotypes carrying the SNCA gene in NHPs showed a variable degree of dopaminergic damage upon the time-dependent aggregation of αSyn (Marmion and Kordower, 2018). In this regard, the use of rAAVs coding for wild-type or mutated forms of αSyn in marmosets showed a variable 30–60% loss of dopaminergic cells together with a well-established αSyn neuropathology (Kirik et al., 2003). Furthermore, when using rAAV5 instead of rAAV2 for triggering αSyn aggregation in marmosets, a constant cell loss above 40% was observed in the injected marmosets (Eslamboli et al., 2007). Considering Old World monkeys (*Macaca fascicularis*), the use of chimeric rAAV1/2 coding for mutated human αSyn resulted in a 50% reduction of tyrosine hydroxylase positive neuron numbers in the substantia nigra 17 weeks post-AAV delivery (Koprich et al., 2016). Furthermore, a similar approach conducted by the delivery of rAAV9-SynA53T into the SNpc of *Macaca fascicularis* reported up to 40% of dopaminergic cell loss with a follow-up of 12 weeks (Sucunza et al., 2021). The use of different methodologies, including the choice of rAAV serotypes and promoters, viral titration, number of injections and follow-up period, may account for the observed differences in terms of neuronal death.

## 6 Viral vectors for therapeutic purposes

The successful development of novel therapeutics for neurodegenerative disorders overall, and for PD in particular, requires fulfilling three important demands: (1) the therapeutic candidate needs to enter the brain (e.g., adequate passage through the blood-brain barrier), (2) any given product needs to reach the desired target in a specific way (e.g., without the need for reaching the entire CNS), and (3) the concentration of the therapeutic product within the targeted structure needs to be high enough for producing the desired effect. By taking these demands all together, it is without doubt that the intraparenchymal delivery of viral vectors carrying a given therapeutic gene can be taken as the most appropriate strategy to follow. Indeed, the use of viral vectors for the treatment of neurodegenerative disorders is a good example of translation of pre-clinical evidence towards clinical trials, beginning with earlier attempts (Flotte and Carter, 1995; Wagner et al., 1999) up to a quickly expanding list of ongoing clinical trials.

One of the key issues driving potential clinical success is represented by the adequate choice of the delivery route for a given gene therapy product. In this regard, macaques are best suited for these experimental designs, bearing in mind that either intrathecal (intracisternal, lumbar puncture and intracerebroventricular), intraparenchymal or intravenous deliveries can be accommodated in NHPs much easier than in rodents (Pignataro et al., 2018). Indeed, regarding intraparenchymal routes of administration and when compared to rodents, several unique anatomical features of NHPs represent a clear benefit. For instance, considering the NHP striatum (i.e. both caudate and putamen), each of these nuclear components are fully parenchymal structures, separated from each other by fiber bundles of the corticospinal tract. By contrast, the caudate and putamen are merged together within the rodent striatum, and traversed by fibers of the corticospinal tract, a fact representing a clear disadvantage for viral deliveries, bearing in mind that viral vectors are most often avidly taken up and transported by fibers of passage.

At present, there is a quickly growing list of ongoing gene therapy clinical trials for PD, most of them based on intraparenchymal deliveries of AAVs. Ongoing trials can be broadly categorized into (i) dopamine-replacement strategies, (ii) modulation of specific neurotransmitter systems, (iii) controlled release of neurotrophic factors, and (iv) trials intended to correct specific loss-of-function genetic mutations (see Fajardo-Serrano et al., 2021).

In summary, clinical trials under current implementation are based on pre-clinical data gathered from studies conducted in NHP models of PD. In order to increase clinical success, gene therapy pre-clinical trials need to be based on relevant animal models of this neurodegenerative disorder, most likely based on NHP models recapitulating the known neuropathology of the disease to the best possible extent.

## 7 Advancing gene editing technologies in NHPs

Advances in gene editing technology, CRISPR-Cas9 and base-editing methods in particular, are now being extended to macaques and New World primate species (Niu et al., 2014; Sato et al., 2016; Zhou et al., 2019; Wang et al., 2020; Zhang et al., 2020), offering opportunities for generating disease models using NHPs. Due to some unique physiological and anatomical features of primates, NHP disease models provide new possibilities for preclinical efficacy studies for therapeutic approaches (pharmacological and physiological intervention), beyond the current use of NHPs for pharmacokinetics and safety assessments. This is particularly relevant for diseases associated with the brain and the immune system, which have primate-specific features distinct from those of rodents (e.g., the cytokine release syndrome or cytokine storm exists in immunotherapy of human and NHPs (Taraseviciute et al., 2018). To generate NHP models of disease associated with known monogenic mutations and multiple susceptibility gene variants, knockout and knock-in technology can now be applied, either in early embryos *in vitro* (Niu et al., 2014; Sato et al., 2016; Zhou et al., 2019; Cui et al., 2018; Yao et al., 2018) or directly in adult tissues *in vivo* (Li et al., 2021). Moreover, in preparation for future gene therapy in humans, NHPs offer an ideal platform for identifying off-targets of gene editing and for developing methods for their elimination. As animal models for preclinical studies, NHPs lack the uniform genetic background that characterizes the highly inbred lines of mouse disease models. To remedy this deficiency, cloning of NHPs by somatic cell nuclear transfer is now feasible (Liu et al., 2018; Liu et al., 2019), although the efficiency of this approach needs to be greatly improved.

## 8 Understanding brain-systems and their self-organization processes

The NHP-brain-related technologies and research-lines described above, such as the viral vector approaches and the advanced gene-editing methods, implicitly assume that changes in the structural and functional organization of the brain - following any kind of intervention - can be well enough defined to permit quantitative descriptions of states and their evolution, which in turn can be used to understand robust and characteristic deviations from “default” normal patterns of brain networks. For instance, the effects of gene editing, targeting the simulation of a psychiatric disorder, would be assessed and further optimized by a scrutiny of potential behavioral modifications, but they should also be evaluated concerning changes in the topology and dynamic connectivity of brain networks, likely corresponding to evolving dynamic brain states. If so, however, an arising thorny question is: What is a brain state to begin with? Can it be quantitatively defined? Which network components should be taken into account for describing the system's collective behavior at a given time?

States of an organism, such as sleep, wakefulness, aphasia or attention have been intensively studied for years, but our understanding and quantitative definition of the actual state of brain-networks possibly related to organism-states remains poor, in spite of all developments in systems neuroscience. This should be hardly surprising, as brains are characterized by a vast number of elements, ultra-high structural complexity, and massive connectivity, all of which change and evolve in response to experience. Information related to sensors and effectors is processed in both parallel and recurrent hierarchical fashions. The connectivity between different hierarchical levels is most often bidirectional, and its specificity and effectiveness are continuously controlled by differentially specific thalamo-associational and neuromodulatory centers. Typically, any observed brain activity is probabilistic, and its evolution initial-condition dependent, with the latter – not surprisingly – reducing regularity, by increasing the statistical randomness observed in systematic analytical and computational brain-studies (e.g., Breakspear, 2017; Tononi et al., 1994). In mathematical physics, such structures are termed *adaptive* Complex Dynamic Systems (CDS), with the term “complex” not meaning “complicated”. Instead, it implies that the behavior of the whole is “emerging”, and it cannot be reduced to, or predicted from, the activity of the system's components, e.g., see Kelso (1995).

Systems such as earthquakes, volcanic eruptions, weather/climate evolution, social communication, market crashes, or genetics and epidemics have long been studied intensively using this CDS approach, and these studies have undoubtedly advanced our ability to predict “random-looking” evolution paths. In contrast, the application of CDS in systems neuroscience has thus far been more limited. It is mostly encountered in human studies using neuroimaging techniques, such as fMRI, diffusion tensor imaging (DTI), magnetoencephalography (MEG) and electroencephalography (EEG) (Bullmore and Sporns, 2009). However, the extensive NHP research and computation modelling of DiCarlo and colleagues has made use of deep, non-linear networks to gain a good understanding of the complex transformations necessary for object recognition (DiCarlo et al., 2012; Hung et al., 2005; Li and DiCarlo, 2010; Rajalingham et al., 2018; Zhuang et al., 2021).

Certainly, in future research, optimizing and applying the CDS methodologies in various experimental animals, including rodents and primates, will gradually provide us with insights into the fundamental and evolutionary preserved self-organization processes of neural networks, including principles of characterization of brain-states and their initial-condition sensitive, unfolding-paths. CDS theories would consider various cognitive capacities as a probabilistic outcome of the interaction of processes at many levels and many systems, including subcortical structures, cortical regions and areas, and various neuromodulatory centers. In principle, even in one species, two entirely different cognitive capacities may reflect the involvement of exactly the same number of cortical and subcortical sites, potentially with similar regional, local responses, but their self-organization and the emerging inter-structure interactions may lead to entirely different behaviors. In other words, behaviors may diﬀer from each other not because of diﬀerences in the active neural sites, but because of diﬀerences in how these sites depend on and aﬀect one another. And this is greatly magnified when the number of potential responses (repertoire) to an intrinsic or environmental event is substantially greater in one species than the other. Thus, if such knowledge can then be applied to animal models of human diseases, the probability to understand the processes underlying brain malfunctions is obviously higher, if the models are NHPs.

Yet, as mentioned above, neural activity in such cases can only be indirectly and imprecisely estimated, often with low spatiotemporal resolution, and occasionally, e.g. in fMRI, reflecting changes in overall non-causal metabolic energy demands, rather than collective activity of neurons within microcircuits and across large-scale systems. Moreover, such an approach, among others, requires also a profound understanding of the neural basis of the brain's structure-specific hemodynamic responses, as well as of the mathematical models permitting the approximation of neural signals from the fMRI time series. Classic system identification techniques fall short when dealing with complex ensembles, such as those comprised of neural, glial, and vascular components (Figley and Stroman, 2011; Mederos et al., 2018; Petzold and Murthy, 2011). More so, when the neurovascular ensemble, including astrocytes and pericytes of some brain structures appears to have feedback loops (e.g., partial modulation of neuronal assemblies by vessel-controlling astrocytes), fall into the category of “non-causal” systems.

Evidently, the aforementioned complexity can be best studied and understood by means of multimodal methodologies. In successfully combined neurophysiology-neurochemistry and fMRI experiments, one can fathom into the neural origin of the up- and down-modulation of imaging patterns by directly recording neurotransmitter and neuromodulator concentrations, as well as into the activity of single neurons, microcircuits, and columns. Such an approach, in turn, permits the estimation of complex-network measures, such as hubs, centralities, connectivity path-lengths, and modularity based on hierarchical clustering, all providing a realistic assessment of brain wide states.

However, neuronal nets in different species develop under different constraints, and comparing interareal and interstructure cortical networks across brains of different size and mammalian order can only provide reliable information on evolutionarily preserved features. As noted above, the number of sensory or motor cortical areas may strongly be species-dependent, connectivity via associational cortical areas or subcortical structures, including thalamic nuclei, may greatly vary, and so can various structural patterns, such as the replication principle in primates, related to the cortico-pulvinar-cortical connections spatially mimicking areal cortico-cortical connections (Shipp, 2003).

In conclusion, multimodal, multiscale and multidisciplinary basic research in primates, investigating the self-organization processes of neural networks, is the only realistic strategy for dealing effectively with a large number of serious neurological and neuropsychiatric disorders. When a crucial problem emerges, the probability of facing it effectively depends to some extent on the difficulty of the problem, but even more importantly and strongly on existing background knowledge that would permit us to construct a meaningful strategic plan. This “background” information, in particular in neuroscience, is typically enormously diverse, and all relevant findings are usually the product of previous, curiosity-driven basic research.

Important for systems neuroscience is also the development of technologies permitting long-term recordings at different spatiotemporal scales. An example is the current microendoscopic calcium imaging (MCI) (Bollimunta et al., 2021), that can be used to track changes before and after gene-editing. MCI, already applied to NHPs, enables recordings of Cellular-Resolution Calcium Dynamics from large populations of neurons simultaneously in more than one cortical site. Most importantly, MCI is stable over several months, allowing the longitudinal tracking of individual neurons and monitoring of their relationship to behavior over long time periods. Integrating MCI into the aforementioned multidisciplinary methodologies will be critically important for projects involving, for instance, genetic engineering, by allowing the observation of subpopulations of neurons before and after genetic changes.

## 9 Current caveats

As indicated, NHPs are a special resource in neuroscience and biomedical research. Scientists and personnel working with NHPs require extensive training and dedication to develop expertise in the specific research techniques and in NHP handling and their ethical and welfare concerns. Arguably, an individual research group does not have all the necessary expertise in-house. Therefore, collaborations are vital to successful future endeavors in NHP neuroscience and biomedical research. Of course, there are many benefits when working collaboratively, including on a global stage – sharing of knowledge, data, and expertise, leading to the potential capability to answer scientific questions faster. However, there are currently caveats to overcome so that global collaborations involving NHPs models may occur. First, unlike human biomedical research which adheres to the World Medical Assocation Declaration of Helsinki, there are no universally accepted and consistent regulatory, or ethical frameworks governing neuroscience and biomedical research involving NHPs. Instead, individual institutions, states, and countries ascribe their own ethical standards and regulations with several institutions around the world adopting the NIH guidelines stipulated in the Guide for the Care and Use of Laboratory Animals (https://grants.nih.gov/grants/olaw/guide-for-the-care-and-use-of-laboratory-animals.pdf) while in Europe, countries adhere to the EU Directive 2010/63/EU. Many neuroscientists involved in NHP research studies are calling for the possibility of a common set of ethical and welfare standards that is internationally acceptable and can lead to international collaborations involving NHP models for the benefit of the monkeys, the scientists, the regulators, the funders, and ultimately the public (Mitchell et al., 2021; Petkov et al., 2022). Similarly, global oversight in the form of an international consortium is proposed for biomedical research involving genetically modified monkeys to ensure ethical regulations, NHP welfare, and appropriate training standards are met (Feng et al., 2020). Critically though, these endeavors must ensure that their mandate is science-led, rather than political.

In this way, new initiatives involving international collaborations between NHP neuroscientists may occur. For example, PRIMatE Data and Resource Exchange (PRIME-DRE; Messinger et al., 2021), which involves the sharing of retrospectively collected NHP neuroimaging data, and newly developed NHP analysis methods and techniques (PRIMatE Data, 2021).

In addition to highlighting the critical need for internationally agreed upon ethical regulations and welfare standards (e.g., cage size and social housing), it must also be highlighted that researchers currently face further unprecedented challenges when working with NHPs for neuroscientific and biomedical research. There are three areas of particular concern, including (1) the transportation of research animals; (2) the ongoing demands from antivivisectionist groups that all animal research must stop; and (3) the export ban of NHPs from China which began during the global pandemic, 2019–2020.

First, safe and reliable transportation of research animals by air, rail, road or sea is an essential element for medical and scientific advancements across the globe. For researchers and institutions that rely on the transportation of laboratory animals, either from one country or continent, to another, or from a breeder to a research institution and for the companies that breed them, the ability to do so is under severe threat. Activist groups opposed to animal research have targeted airlines in a concerted effort to harass, intimidate and extort companies to hinder the research process. Regrettably, they have been successful in doing so with only a few transport providers still willing to transport research animals. Whilst opponents of animal research have sought to halt the transport of all laboratory research animals, it is the transport of NHPs and canines that is the main focus of their campaigns. Air France (the only commercial airline that were transporting NHPs) announced in July 2022 that they would no longer transport NHPs used in scientific research. In Europe and in the USA, both the transport and the breeding of canines, “purpose-bred” for biomedical research face insidious and disruptive activist campaigning. Medical research and scientific understanding are a global collaborative effort. Without the ability to move research animals crucial to scientific studies, the search for new treatments will be disrupted.

Second, although numbers of NHPs are relatively low in neuroscience and biomedical research (currently 0.09% of all animal use – https://www.eara.eu/animal-research-statistics EU statistics 2020), NHP models have been integral in the development of many scientific and biomedical advances. If campaigns had been successful in the 1980s in halting the transportation of primates, we would be lacking many advances in scientific understanding involving NHPs over the past 30 years, including, but not limited, to the apomorphine treatment for PD (Luquin et al., 1993); the development of antiretroviral therapy for the treatment of HIV/AIDS (Tsai et al., 1995) surgical treatment for macular degeneration (Tan et al., 2021; Valentino et al., 1995) and new techniques in stroke rehabilitation therapy (Higo, 2021). It may be the case that if activist groups succeed in halting the transportation of NHP or canines, they may be emboldened to move onto other animal models.

Finally, the current stop to the export of NHPs from China has resulted in a global shortage of NHPs used for scientific purposes. China is the world's largest exporter of NHPs. However, since January 2020, NHP researchers in Europe, North America, and other continents have been unable to access the animals they need, which hampers innovation and discovery. In relation to Europe, as governments seeks to strengthen resilience in health care systems and innovation throughout the EU, this restriction on NHP availability creates obstacles to research and improved public health. This is particularly true in the context of the pandemic, given that all Covid-19 vaccines (including Germany's BioNTech, Pfizer vaccine) approved in Europe have relied on testing in NHPs to demonstrate safety and efficacy. Further Covid-19 vaccines and therapeutics continue to be developed in response to new Covid-19 variants and will continue to depend on access to NHPs for safety and efficacy testing. In 2022, the European Commission has undertaking a feasibility study on the supply of NHPs into the EU. The study sort to establish what progress is being made towards the supply of animals for scientific research that only come from self-sustained colonies (SSC). Article 10 and Annex II of Directive (2010)/63/EU on the protection of animals used for scientific purposes states that, after an appropriate transition period (from November 2022) only NHPs, who are the offspring of animals bred in captivity, will be used in research. This is defined as animals which are F2 (filial 2) generation or above, or NHPs sourced from SSC. In 2017, a feasibility study concluded that sufficient progress was being made to phase out the use of wild caught and F1 (filial 1) animals, and that it would therefore be possible to use only F2, or NHPs from SSC, by 2022. However, the global landscape has changed dramatically since 2017, with unprecedented events meaning that this is no longer the case. Since the Article 10 feasibility study in 2017, unprecedented global changes have impacted on both the supply and demand for NHP. While the biomedical research community support the plan to move to SSC in principle, it is clear that the current shortage in NHPs for research would only be exacerbated by an F1 restriction. At present, it is not possible to predict a time when it will be feasible to prohibit the use of F1 animals, without having a detrimental impact on life sciences, innovation and public health in Europe.

Additionally, recent scientific findings indicate that NHPs coming from SSC may at times show abnormal brain development and function due to genetic defects (Bridge et al., 2019), which would compromise all research with these animals. A ban on importing NHPs would also hamper the development of transgenic lines and would force researchers to develop these lines at multiple places in the world creating unnecessary duplication. Further restrictions on the ability of European researchers to access NHPs needed for research are likely to increase pressures to shift NHP neuroscience research out of Europe.

## 10 Conclusions

The scientific evolutions outlined above are undoubtedly very positive because they indicate that better techniques will help us to better understand and treat the brain, and – even more importantly – that the large molecular neuroscience field is slowly but steadily moving towards human applications, which will ultimately transform the diagnosis and treatment of patients with brain disorders. At this moment in time, all evidence indicates that NHPs will continue to play a crucial role in the development of better medical care for patients, as they have done in the past in neuroscience and numerous other domains of medicine (Buffalo et al., 2019; Bushmitz, 2014; Friedman et al., 2017). In order to have better medical care and viable treatments in the foreseeable future and beyond, it is essential that biomedical researchers working with NHP models have the continued support of governments, funders, policymakers, and the public so that all of us may benefit from the knowledge produced from these invaluable NHP research models.

## CRediT authorship contribution statement

**Peter Janssen:** Conceptualization, Writing – original draft, Writing – review & editing. **Tadashi Isa:** Writing – original draft, Writing – review & editing. **Jose Lanciego:** Writing – original draft, Writing – review & editing. **Kirk Leech:** Writing – original draft, Writing – review & editing. **Nikos Logothetis:** Writing – original draft, Writing – review & editing. **Mu-Ming Poo:** Writing – original draft, Writing – review & editing. **Anna S. Mitchell:** Conceptualization, Writing – original draft, Writing – review & editing.

## Declaration of competing interest

The authors declare that they have no known competing financial interests or personal relationships that could have appeared to influence the work reported in this paper.
