# Supplementary material for: Visualizing advances in the future of primate neuroscience research
Source: Curr Res Neurobiol. 2022 Dec 13;4:100064. doi: 10.1016/j.crneur.2022.100064 (PMC9792703; doi:10.1016/j.crneur.2022.100064)
Supplement: Multimedia component 1 [file mmc1.pdf]

# Peer Review Overview

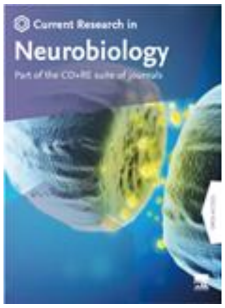


**Manuscript Title:** Visualizing advances in the future of primate neuroscience research


| | |
|---|---|
| Received | Feb 17, 2022 |
| 1st Decision | Apr 10, 2022 |
| 1st Revision Submitted | Sep 30, 2022 |
| Accepted | Nov 24, 2022 |


---

## 1st Decision letter

**Reference:** CRNEUR-D-22-00010
**Title: :** Visualizing advances in the future of primate neuroscience research
**Journal:** Current Research in Neurobiology

Dear Dr. Mitchell,

Thank you for submitting your manuscript to Current Research in Neurobiology.

The reviewers recommend reconsideration of your manuscript following minor revision and modification. I invite you to resubmit your manuscript after addressing the comments below. Please resubmit your revised manuscript by .

When revising your manuscript, please consider all issues mentioned in the reviewers' comments carefully: please outline every change made in response to their comments and provide suitable rebuttals for any comments not addressed. Please note that your revised submission may need to be re-reviewed.

Current Research in Neurobiology values your contribution and I look forward to receiving your revised manuscript.

*CRNEUR* aims to be a unique, community-led journal, as highlighted in the Editorial Introduction. As part of this vision, we will be regularly seeking input from the scientific community and encourage you and your co-authors to take the survey.

Kind regards,

Christopher I. Petkov
Editor in Chief
Current Research in Neurobiology

## Comments from Editors and Reviewers:

This manuscript was well received by all three reviewers. The feedback received from the reviewers is constructive and will help to finesse a couple points further in the manuscript. There are recommendations to improve the 'Complex Dynamic Systems' section, a consideration of manuscript title and the scope of such title, along with a few references suggested by the reviewers to consider adding to the current manuscript.

**Reviewer #1:**

This is an excellent and timely review of the prospects for working with NHPs to solve major questions in basic and applied neuroscience. I have relatively few comments and most of these serve to encourage the authors in their efforts

page 6 "many researchers nowadays study the visual cortex in mice instead of in NHPs"

It seems to me that this is a weak example to quote here. The more we understand about mouse vision, the more divergent from primate vision it seems to be. One may cite the peculiar forms of binocular co-ordination, the constant thrum of what is apparently motor-based activity in the mouse visual cortex and the considerable differences in columnar structures as recent findings. Added to the known differences in color vision, there do seem to be substantial differences in functional organization between rodents and primates.

Page 6 "the large brains of NHPs with sulci and gyri similar to the human brain,"

This is only true of Old World monkeys.

Page 6 "testing of new life-saving medicines"
There is no need for them to be "life-saving" in order to justify investigation in NHPs. Whilst no-one would want research for frivolous reasons, there are a number of chronic conditions that are disabling without being immediately life-threatening. Quality of life can support an ethical case for use of NHPs

Page 13: complex dynamic systems

This commentary is valuable but whilst it made the case for studying animal models of human brain function very clearly, it did not have quite the same forcefulness in respect of making a case that the CDS insights would be uniquely advanced using the brains of NHPs. There was an attempt based on the number of cortical areas on page 14, but this did not deliver reasons why CDS phenomena are evident within the brains of NHPs but not mice.

Page 15
The call for an international consortium to maintain, promote and educate about welfare standards is welcome provided that it is science-led, rather than politics-led. The recent stand-off between the European Commission and the European Parliament in this area of policy formation is a warning to everyone.

Page 17
The comments in the article on page 17, which I assume refer to the possibility of restricting all research

use to F2 animals, are very pertinent here. As the Wikipedia entry on F1 hybrids in agriculture states "When F1 cultivars are used as parents, their offspring (F2 generation) vary greatly from one another". A specific vertebrate example is cichlid fish [1], where there is substantial loss of viability in the F2 generation. Now the colony management for European NHP production does make extensive use of F1 cross breeding to "refresh" the colony at various times. But for political reasons, use of F2 animals in the research lab is therefore the situation that may be faced universally in Europe for NHP work. In the UK the closed colony at Centre for Macaques at Porton Down produces essentially in-bred F2 (or greater) animals for research use. This has produced offspring with naturally occurring defects of brain development and function (see [2]). In fact, there is more than one animal from this colony with this kind of substantial defect. It seems essential not to be tied to specific modes of production for research purposes. F2 animals will often be fine, but with the increasing use of molecular interventions, as correctly endorsed in the article, the stability of the genetic base for the NHPs that are actually used in research procedures becomes a critically important factor.

References

1. Stelkens, R., C. Schmid, and O. Seehausen, Hybrid Breakdown in Cichlid Fish. PLoS ONE, 2015. 10.
2. Bridge, H., et al., Preserved extrastriate visual network in a monkey with substantial, naturally occurring damage to primary visual cortex. eLife, 2019. 8: p. e42325.

**Reviewer #2:**

The manuscript submitted by Janssens et al. is a review on the interest of using non-human primate (NHP) in neuroscience research. It is a very timely and important topic which fits perfectly well within the scope of Current Research in Neurobiology. The manuscript is well-written and provides a convincing demonstration that NHPs still constitute a very good animal model to progress in this research domain. The manuscript covers well different fields (fundamental neuroscience, biomedical research, disease modeling and therapeutics) and in my opinion, it could almost be published in its current form.

My only comment is that it would perhaps be useful to specify a little bit more which species of primate is used in the various works cited. I have the impression that most were probably performed in macaques but some were also realized in marmoset (e.g., in the 'Viral vectors for disease-modeling purposes' section). Given the rising influence of the marmoset model in neuroscience studies, it might also be useful to add a section that would specify in more detail the possibilities offered by this NHP model.

**Reviewer #3:**

The article by Janssen et al. gives insight in the current and future use of non-human primates (NHPs) in neuroscience. The review covers several research topics and indicates that is it likely that NHPs will remain essential for progress in the foreseeable future. The article constitutes an important contribution to the debate about the use of animals in research. I do have several suggestions for improvement, which are listed below.

Major concerns
1) The title suggests that the scope of the article is the use of NHP across all domains of neuroscience. However, the article focuses on several more applied topics and does not consider many advances that are being made in the fundamental understanding in the working of the brain, some of which uniquely depend on NHP research. The article would benefit from a paragraph of scientific progress in higher cognitive functions, such as decision making (e.g. the work of Shadlen et al., Newsome et al.), object recognition (e.g. DiCarlo et al) and higher order planning (e.g. Rushworth et al.). To give another example: the pathways for saccadic eye movements cannot easily be studied in rodents, which use saccadic eye movement in another manner. The alternative would be to further focus the title (and abstract) of the article.
2) The authors might want to address single unit recordings in humans, introduced by Itzak Fried and his collaborators. This method can only be used in a few brain regions.
3) The section on "Complex Dynamical Systems" could be improved. The work of e.g. DiCarlo et al, makes great use of deep, non-linear networks to gain a good understanding of the complex transformations necessary of object recognition. It seems that we have moved on from the days that we need to borrow insights from studies on e.g. earthquakes to make progress in neuroscience.
4) p. 17 It might be useful to also consider the consequences on a ban on importing NHPs in the development of transgenic lines and making them available worldwide. Would this imply that these lines would need to be developed independently at multiple places?

Minor concerns
Background section
- "For example, safety testing of pharmaceuticals and medical devices most often require NHPs because of the poor definition of pharmacokinetic parameters in isolated in vitro systems, and difficulties in extrapolating from in vitro data to humans."
Here the authors may also mention the possible difficulties in extrapolating from non-primate species to humans.

- "utterly noninvasive" could be replaced by "noninvasive"

- (p. 3) In the sections on fMRI limitations the unclear coupling between neuronal activity and the BOLD could be mentioned more clearly

- (p. 4) In the list of new technologies, high density electrophysiology, e.g. as made possible by Neuropixels probes, could be mentioned.

- (p. 4) "foresight of Juan Carlos López". Is there a reference to a report for those who would like to understand what is meant in more detail?

- (p. 4) "testing drug candidates in preclinical stages" could be expanded to other therapeutic intervations (e.g. BCI) here.

Section "NHP models remain fundamental for neuroscience and biomedical research"
- In the list of brain structures where primates differ from rodents, the visual cortex with its hierarchical

structure in primates and the very different structure in rodents could be mentioned.

The title "Novel gene therapy approaches for disease modeling and therapeutics" seems to imply that gene therapy for disease modelling is a form of therapy. This should be rephrased.

"desired efficacious effect" -> "desired effect"

What is a "cytokine storm"? Please explain.

# 1st Author Response Letter

## Response to comments from Editors and Reviewers:

We would like to thank all three reviewers and the Editor for their insightful and supportive comments and their time taken to review our manuscript. We have detailed below how we have incorporated each of the reviewers' points and highlighted the text in the manuscript in red. We believe these comments have helped to improve our manuscript.

**Comments from Reviewer 1**

This is an excellent and timely review of the prospects for working with NHPs to solve major questions in basic and applied neuroscience. I have relatively few comments and most of these serve to encourage the authors in their efforts

Thank you for these supportive comments.

page 6 "many researchers nowadays study the visual cortex in mice instead of in NHPs"

It seems to me that this is a weak example to quote here. The more we understand about mouse vision, the more divergent from primate vision it seems to be. One may cite the peculiar forms of binocular co-ordination, the constant thrum of what is apparently motor-based activity in the mouse visual cortex and the considerable differences in columnar structures as recent findings. Added to the known differences in color vision, there do seem to be substantial differences in functional organization between rodents and primates.

We have adjusted the text on page 8 to include these details.

Further, many researchers nowadays study the visual cortex in mice instead of in NHPs. However, there are limitations in translating results from rodent models to primates given the species differences in visual abilities (e.g., differences in binocular disparity and color spectrum detection), differences in cortical size, and the relative reliance on vision for primates verses other sensory processes in rodents (Graïc et al., 2022; Halley and Krubitzer, 2019; Laramée and Boire, 2015). In addition, biomedical researchers investigating human retinal damage and treatments still require NHP models given the

similarities in the vascular supply and in the retina (e.g. the presence of a fovea as in humans) of NHPs and humans.

Page 6 "the large brains of NHPs with sulci and gyri similar to the human brain,"

This is only true of Old World monkeys.

We have qualified this sentence to indicate it is Old World Macaque monkeys

Page 6 "testing of new life-saving medicines"
There is no need for them to be "life-saving" in order to justify investigation in NHPs. Whilst no-one would want research for frivolous reasons, there are a number of chronic conditions that are disabling without being immediately life-threatening. Quality of life can support an ethical case for use of NHPs

We have deleted life-saving

Page 13: complex dynamic systems

This commentary is valuable but whilst it made the case for studying animal models of human brain function very clearly, it did not have quite the same forcefulness in respect of making a case that the CDS insights would be uniquely advanced using the brains of NHPs. There was an attempt based on the number of cortical areas on page 14, but this did not deliver reasons why CDS phenomena are evident within the brains of NHPs but not mice.

We have provided additional details to support the use of NHPs as detailed below and on page 16

Certainly, in future research, optimizing and applying the CDS methodologies in various experimental animals, including rodents and primates, will gradually provide us with insights into the fundamental and evolutionary preserved self-organization processes of neural networks, including principles of characterization of brain-states and their initial-condition sensitive, unfolding-paths. CDS theories would consider various cognitive capacities as a probabilistic outcome of the interaction of processes at many levels and many systems, including subcortical structures, cortical regions and areas, and various neuromodulatory centers. In principle, even in one species, two entirely different cognitive capacities may reflect the involvement of exactly the same number of cortical and subcortical sites, potentially with similar regional, local responses, but their self-organization and the emerging inter-structure interactions may lead to entirely different behaviors. In other words, behaviors may differ from each other not because of differences in the active neural sites, but because of differences in how these sites depend on and affect one another. And this is greatly magnified when the number of potential responses (repertoire) to an intrinsic or environmental event is substantially greater in one species than the other. Thus, if such knowledge can then be applied to animal models of human diseases, the

probability to understand the processes underlying brain malfunctions is obviously higher, if the models are NHPs.

Page 15

The call for an international consortium to maintain, promote and educate about welfare standards is welcome provided that it is science-led, rather than politics-led. The recent stand-off between the European Commission and the European Parliament in this area of policy formation is a warning to everyone.

This is a very good point to make and we have highlighted this – now on page 19.

Critically though, these endeavors must ensure that their mandate is science-led, rather than political.

Page 17

The comments in the article on page 17, which I assume refer to the possibility of restricting all research use to F2 animals, are very pertinent here. As the Wikipedia entry on F1 hybrids in agriculture states "When F1 cultivars are used as parents, their offspring (F2 generation) vary greatly from one another". A specific vertebrate example is cichlid fish [1], where there is substantial loss of viability in the F2 generation. Now the colony management for European NHP production does make extensive use of F1 cross breeding to "refresh" the colony at various times. But for political reasons, use of F2 animals in the research lab is therefore the situation that may be faced universally in Europe for NHP work. In the UK the closed colony at Centre for Macaques at Porton Down produces essentially in-bred F2 (or greater) animals for research use. This has produced offspring with naturally occurring defects of brain development and function (see [2]). In fact, there is more than one animal from this colony with this kind of substantial defect. It seems essential not to be tied to specific modes of production for research purposes. F2 animals will often be fine, but with the increasing use of molecular interventions, as correctly endorsed in the article, the stability of the genetic base for the NHPs that are actually used in research procedures becomes a critically important factor.

References

1. Stelkens, R., C. Schmid, and O. Seehausen, Hybrid Breakdown in Cichlid Fish. PLoS ONE, 2015. 10.
2. Bridge, H., et al., Preserved extrastriate visual network in a monkey with substantial, naturally occurring damage to primary visual cortex. eLife, 2019. 8: p. e42325.

We have provided further details about this predicament in the text – now on page 21.

Article 10 and Annex II of Directive 2010/63/EU on the protection of animals used for scientific purposes states that, after an appropriate transition period (from November 2022) only NHPs, who are the offspring of animals bred in captivity, will be used in research. This is defined as animals which are F2 (filial 2) generation or above, or NHPs sourced from SSC. In 2017, a feasibility study concluded that sufficient progress was being made to phase out the use of wild caught and F1 (filial 1) animals, and that it would therefore be possible to use only F2, or NHPs from SSC, by 2022. However, the global landscape has changed dramatically since 2017, with unprecedented events meaning that this is no longer the case.

Since the Article 10 feasibility study in 2017, unprecedented global changes have impacted on both the supply and demand for NHP. While the biomedical research community support the plan to move to SSC in principle, it is clear that the current shortage in NHPs for research would only be exacerbated by an F1 restriction. At present, it is not possible to predict a time when it will be feasible to prohibit the use of F1 animals, without having a detrimental impact on life sciences, innovation and public health in Europe.

Additionally, recent scientific findings indicate that NHPs coming from SSC may at times show abnormal brain development and function due to genetic defects (Bridge et al., 2019), which would compromise all research with these animals. A ban on importing NHPs would also hamper the development of transgenic lines and would force researchers to develop these lines at multiple places in the world creating unnecessary duplication.

**Comments from Reviewer 2**

The manuscript submitted by Janssens et al. is a review on the interest of using non-human primate (NHP) in neuroscience research. It is a very timely and important topic which fits perfectly well within the scope of Current Research in Neurobiology. The manuscript is well-written and provides a convincing demonstration that NHPs still constitute a very good animal model to progress in this research domain. The manuscript covers well different fields (fundamental neuroscience, biomedical research, disease modeling and therapeutics) and in my opinion, it could almost be published in its current form.

Thank you for these supportive comments.

My only comment is that it would perhaps be useful to specify a little bit more which species of primate is used in the various works cited. I have the impression that most were probably performed in macaques but some were also realized in marmoset (e.g., in the 'Viral vectors for disease-modeling purposes' section). Given the rising influence of the marmoset model in neuroscience studies, it might also be useful to add a section that would specify in more detail the possibilities offered by this NHP model.

We have now clarified the use of macaques and marmoset models more clearly throughout the text.

**Comments from Reviewer 3**

The article by Janssen et al. gives insight in the current and future use of non-human primates (NHPs) in neuroscience. The review covers several research topics and indicates that is it likely that NHPs will remain essential for progress in the foreseeable future. The article constitutes an important contribution to the debate about the use of animals in research. I do have several suggestions for improvement, which are listed below.

Thank you for these supportive comments.

Major concerns

1) The title suggests that the scope of the article is the use of NHP across all domains of neuroscience. However, the article focuses on several more applied topics and does not consider many advances that are being made in the fundamental understanding in the working of the brain, some of which uniquely depend on NHP research. The article would benefit from a paragraph of scientific progress in higher cognitive functions, such as decision making (e.g. the work of Shadlen et al., Newsome et al.), object recognition (e.g. DiCarlo et al) and higher order planning (e.g. Rushworth et al.). To give another example: the pathways for saccadic eye movements cannot easily be studied in rodents, which use saccadic eye movement in another manner. The alternative would be to further focus the title (and abstract) of the article.

We have included three additional paragraphs to highlight these important contributions on pages 6-7

We have learned a great deal from neuroanatomical, neurophysiological, direct electrical- and optical-stimulation, molecular and lesion studies in NHPs (mainly macaques). Along with other species, primates were also routinely used to develop and test treatments for diseases and medical conditions. Examples include the polio vaccine, insulin treatments, safer techniques for heart, eye, and bone surgeries, substantially better medical care for prenatal and postnatal infants, treatments for polycystic ovary syndrome and endometriosis in women, discovery of the Rh blood incompatibility in infants and mothers, and many more (see, for instance, MedlinePlus and sites of National Primate Research Centers, e.g. https://nprcresearch.org/primate/).

Yet, translational NHP studies have not been the only type of research to contribute to medical progress. Fundamental basic, curiosity-driven research substantially improved our understanding of brain function and dysfunction. A striking example is the brain research that preceded so-called Constraint-Induced Movement Therapy (CIMT) that was developed in NHPs by Edward Taub (Taub et al., 2002). Deep Brain Stimulation (DBS) used in the treatment of patients with Parkinson's Disease (PD) is another example of basic research influencing an applied medical one. The method was developed from research on macaques (Rosenow et al., 2004). The background information collected by curiosity driven, basic research is of fundamental importance for any rising question in any applied research field. Moreover, basic research in primates offered the first insights into the hierarchical organization of the visual system, each stage of which consists of different areas or modules that work - to some extent - parallel to each other, analyzing different visual attributes (Felleman and Van Essen, 1991; Hubel, 1988; Livingstone and Hubel, 1988; Zeki, 1993; Zeki, 1998). Selective damage to different areas demonstrated that the dorsal (parietal cortex) stream processes information about the location and motion of objects, while the ventral (temporal cortex) stream processes information related to object recognition (Mishkin et al., 1983; Ungerleider & Mishkin, 1982).

Neurophysiological studies in the NHP have also offered insights into the neural underpinnings of the processes of attention, decision making (Desimone and Duncan, 1995; Gold and Shadlen, 2007; O'Connell et al., 2018; Sugrue et al., 2005), higher order planning (Rushworth and Behrens, 2008), conscious perception of multistable objects (Logothetis, 1999), and object recognition (DiCarlo and Cox, 2007; DiCarlo et al., 2012; Logothetis, 1998). Remarkably, non-human primates were also the main source of information related to cognitive capacities, usually associated only with humans, including cultural transmission, and the origin of language (Lloyd, 2004). Overall, NHP studies like those very briefly described above, offer true insights into the cognitive capacities of humans, likely also because of the anatomical and functional similarities of the two species.

2) The authors might want to address single unit recordings in humans, introduced by Itzak Fried and his collaborators. This method can only be used in a few brain regions.

We have included these studies on page 5.

Yet, invasive studies in humans remain rare (e.g., Quiroga et al., 2005; Hochberg et al., 2006), and although in recent years more human cortical areas have been explored (Aflalo et al., 2015; Decramer et al., 2019), the number of areas that can be recorded in humans remains extremely limited.

3) The section on "Complex Dynamical Systems" could be improved. The work of e.g. DiCarlo et al, makes great use of deep, non-linear networks to gain a good understanding of the complex transformations necessary of object recognition. It seems that we have moved on from the days that we need to borrow insights from studies on e.g. earthquakes to make progress in neuroscience.

We have updated this section and included some of the DiCarlo references and provided some thoughts on how CDS could be used in further systems neuroscience research on page 16. Reviewer 1 also included comments to request and extension of the CDS analyses for NHP studies – please see this additional detail above and on pages 16-17.

Systems such as earthquakes, volcanic eruptions, weather/climate evolution, social communication, market crashes, or genetics and epidemics have long been studied intensively using this CDS approach, and these studies have undoubtedly advanced our ability to predict "random-looking" evolution paths. In contrast, the application of CDS in systems neuroscience has thus far been more limited. It is mostly encountered in human studies using neuroimaging techniques, such as fMRI, diffusion tensor imaging (DTI), magnetoencephalography (MEG) and electroencephalography (EEG) (Bullmore and Sporns, 2009). However, the extensive NHP research and computation modelling of DiCarlo and colleagues has made use of deep, non-linear networks to gain a good understanding of the complex transformations

necessary for object recognition (DiCarlo et al., 2012; Hung et al., 2005; Li and DiCarlo, 2010; Rajalingham et al., 2018; Zhuang et al, 2021).

4) p. 17 It might be useful to also consider the consequences on a ban on importing NHPs in the development of transgenic lines and making them available worldwide. Would this imply that these lines would need to be developed independently at multiple places?

We have updated this section with some possible scenarios – now on page 21.

A ban on importing NHPs would also hamper the development of transgenic lines and would force researchers to develop these lines at multiple places in the world creating unnecessary duplication.

Minor concerns
Background section
- "For example, safety testing of pharmaceuticals and medical devices most often require NHPs because of the poor definition of pharmacokinetic parameters in isolated in vitro systems, and difficulties in extrapolating from in vitro data to humans."
Here the authors may also mention the possible difficulties in extrapolating from non-primate species to humans.

We have done this on page 3

Animal models (including NHPs) are not able to encapsulate a complete disease state present in humans, so there remain some difficulties in extrapolating from animal models to humans as well.

- "utterly noninvasive" could be replaced by "noninvasive"

Changed

- (p. 3) In the sections on fMRI limitations the unclear coupling between neuronal activity and the BOLD could be mentioned more clearly

We have explained this further on page 4-5

To better understand the relation of the BOLD imaging signal to its underlying neural events, we briefly review the nature of the neurophysiological signal commonly reported from invasive animal studies (for extensive reviews see Logothetis, 2008; Logothetis & Wandell, 2004). The comprehensive (with a bandwidth of 0.05Hz through 23-30kHz) extracellular field potential measured in intracranial recordings captures at least three different types of activity: single-unit activity representing the action potentials of well isolated neurons next to the electrode tip; multiple unit activity (MUA) reflecting the spiking of small neural populations in a sphere of 100–300 μm radius; and perisynaptic activity of a neural population within 0.5–3mm of the electrode tip, which is reflected in the variation of the low-frequency components

of the extracellular field potential. MUA and local field potentials (LFPs) can be reliably segregated by frequency band separation. The frequency range of 800–3,000 Hz is used in most recordings to obtain MUA; a low-pass filter cutoff of approximately 250 Hz is used to obtain LFP. A large number of experiments have presented data indicating that such a band separation does indeed underlie different neural activities. Thus, their relationship to the BOLD imaging signal is not only able to provide us with insights into the mechanisms underlying the hemodynamic changes, it can also help us with a more detailed interpretation of the functional significance of the activation patterns observed in MR imaging.

Combined physiology-fMRI experiments showed that the BOLD imaging signal predominantly reflects regional *perisynaptic* activity, which includes the classical events of synaptic transmission with its respective population of excitatory or inhibitory postsynaptic potentials, as well as a number of integrative processes, including somatic and dendritic spikes with their ensuing afterpotentials, and voltage-dependent membrane oscillations. These events represent the regional modulation, processing and elaboration of incoming signals, and correlate largely with changes in the LFPs. In other words, the LFPs reflect effects such as neuromodulation, the influence of "background" barrages of synaptic potentials on excitation-inhibition balance, and interactions between interneurons and pyramidal cells, all of which might act as determinant factors for the ensuing hemodynamic response (Logothetis, 2008). As long as spiking and LFP activity resemble each other (e.g., during feedforward propagation of sensory signals), the BOLD response appears to be strongly correlated with both signals. Yet a striking, undiminished hemodynamic response may often be observed in cases in which neuronal firing is entirely absent despite a clear and strong stimulus-induced modulation of the field potentials (Mathiesen et al., 1998; Rauch et al., 2008; Viswanathan and Freeman, 2007). Such results were later confirmed in a series of experiments using either mass-univariate linear and nonlinear methods (Lippert et al., 2010; Ludtke et al., 2010; Zappe et al., 2008), or multivariate techniques (Biessmann et al., 2010; Murayama et al., 2010).

Despite its shortcomings, fMRI is an outstanding tool for gaining insights into network activity, and it can often reveal activated regions that may be missed by electrical recordings. Activation of populations whose cells have a close-field (low dipole moment) arrangement may occasionally be missed both in the LFP and MUA signals; yet changes in energy metabolism and subsequent changes in hemodynamics may still impact the fMRI signal. Importantly, activations during imaging are always neurogenic and maps of activity reveal active networks, regardless of whether they reflect sensory or modulatory signals (Logothetis, 2010). Interpreting and understanding neuroimaging signals requires complementary invasive neuroscience research.

- (p. 4) In the list of new technologies, high density electrophysiology, e.g. as made possible by Neuropixels probes, could be mentioned.

We have included this phrase and a reference on page 5.

and high density electrophysiology, e.g., as made possible by Neuropixels® probes (Steinmetz et al., 2021).

- (p. 4) "foresight of Juan Carlos López". Is there a reference to a report for those who would like to understand what is meant in more detail?

We have included this reference now on page 6 and in the reference list.

- (p. 4) "testing drug candidates in preclinical stages" could be expanded to other therapeutic intervations (e.g. BCI) here.

We have included this phrase on page 6.

Section "NHP models remain fundamental for neuroscience and biomedical research"
- In the list of brain structures where primates differ from rodents, the visual cortex with its hierarchical structure in primates and the very different structure in rodents could be mentioned.

Reviewer 1 also suggested this and it has been incorporated as detailed above on page 8.

The title "Novel gene therapy approaches for disease modeling and therapeutics" seems to imply that gene therapy for disease modelling is a form of therapy. This should be rephrased.

This subheading has been changed to Novel gene therapy approaches that model diseases and therapeutics

"desired efficacious effect" -> "desired effect"

Changed

What is a "cytokine storm"?

We have clarified this on page 14 to read the ‘cytokine release syndrome or cytokine storm’. The meaning of the cytokine release syndrome occurs when your immune system reacts more strongly to immunotherapy drugs or to infection than it should.

## Accept Letter

Dear Dr. Mitchell,

Thank you for submitting your manuscript to Current Research in Neurobiology.

I am pleased to inform you that your manuscript has been accepted for publication.

My comments, and any reviewer comments, are below.

Your accepted manuscript will now be transferred to our production department. We will create a proof which you will be asked to check, and you will also be asked to complete a number of online forms required for publication. If we need additional information from you during the production process, we will contact you directly.

We appreciate and value your contribution to Current Research in Neurobiology. We regularly invite authors of recently published manuscript to participate in the peer review process. If you were not already part of the journal's reviewer pool, you have now been added to it. We look forward to your continued participation in our journal, and we hope you will consider us again for future submissions.

*CRNEUR* aims to be a unique, community-led journal, as highlighted in the Editorial Introduction. As part of this vision, we will be regularly seeking input from the scientific community and encourage you and your co-authors to take the survey.

We would also like to invite you to take part in our CRNEUR Author Question & Answer (Q&A), which could get published alongside your article and help to promote it. We suspect you might have an interesting story of perseverance or team work that was required for the research study to complete, or a diversity of perspectives that you might share, as a way of inspiring others about neuroscience.

Kind regards,

Christopher I. Petkov
Editor in Chief
Current Research in Neurobiology

Editor and Reviewer comments:

Reviewer #3 raised a couple of good points, one being whether the manuscript might be stronger without the paragraph on complex dynamical systems or if this paragraph should be revised accordingly. There is also a note on updating the sentence related to Air France as an airline that still transports animals. Reviewer #3 raised the point that the situation has changed - it appears the airline announced in summer 2022 that they will cease to transport animals.

Reviewer 1: I am satisfied with all the amendments, which are helpful.

Reviewer 2: The authors responded well to my previous comments and I believe the manuscript is now ready for publication. Congratulations for this work.

Reviewer 3: The Ms has been improved and is ready for publication. It is an important contribution to the field.

There is one section on complex dynamical systems that is a bit vague and speculative. I don't think that earthquakes are very relevant to neuroscience. The paper would be even better without this section, but if the authors want to keep it, the Ms is still very worthy of publication.

One note: Air France/KLM have, in the meantime, also stopped transporting NHP, so this sentence should be updated.

*-------- End of Review Comments --------*
